# Podocalyxin Promotes Glioblastoma Multiforme Cell Invasion and Proliferation via β-Catenin Signaling

**DOI:** 10.1371/journal.pone.0111343

**Published:** 2014-10-28

**Authors:** Yu Liu, Yu-Gang Jiang

**Affiliations:** Department of Neurosurgery, The Second Xiangya Hospital, Central South University, Changsha, China; Faculty of Medicine & Health Sciences, United Arab Emirates

## Abstract

Both podocalyxin (PODX) and β-catenin (β-cat) signaling reportedly play important roles in glioblastoma multiforme (GBM) progression. In this study, we for the first time explored crosstalk between PODX and β-cat signaling in GBM cells, and assessed its impact on GBM cell invasion and proliferation. Stable overexpression of PODX in LN-229 and U-118 MG human GBM cells increased the soluble/intracellular β-cat level, TOPflash luciferase reporter activity, the mRNA levels of β-cat signaling target genes, matrix metalloproteinase 9 (MMP9) expression/activity, and cell invasion and proliferation, which was abolished by selective p38 mitogen-activated protein kinase (MAPK) inhibitor PD169316 and selective β-cat signaling inhibitor CCT031374. On the other hand, stable knockdown of PODX in LN-229 and U-118 MG cells decreased the soluble β-cat level, TOPflash luciferase reporter activity, the mRNA levels of β-cat signaling target genes, MMP9 expression/activity, and cell invasion and proliferation, which was completely reversed by overexpression of a constitutively active β-cat mutant. In addition, overexpression of PODX induced p38 MAPK activity and inactivating phosphorylation of glycogen synthase kinase-3β (GSK-3β) at serine 389 in LN-229 and U-118 MG cells, which was abolished by PD169316, but not CCT031374; knockdown of PODX decreased p38 MAPK activity and inactivating phosphorylation of GSK-3β at serine 389 in both cell lines, which was not significantly affected by overexpression of constitutively active β-cat. In conclusion, this study indicates that PODX promotes GBM cell invasion and proliferation by elevating the soluble β-cat level/β-cat signaling through the p38 MAPK/GSK-3β pathway. Uncovering the PODX/β-cat signaling axis adds new insights not only into the biological functions of PODX and β-cat, but also into the molecular mechanisms underlying GBM progression.

## Introduction

Glioblastoma multiforme (GBM) is by far the most common and most malignant primary adult brain tumor [1]. Despite great advances in surgery, chemotherapy and radiotherapy, the median survival is only 12 to 15 months for patients with GBM [2]. The poor prognosis of GBM is largely attributed to their rapid growth, invasiveness, and high rate of recurrence [3]. The highly invasive nature of GBM makes surgical resection non-curative, and it has also been proposed that invading cells may be more resistant to radiation and chemotherapy [3]. Therefore, it is important to identify and confirm potential therapeutic targets involved in the invasion and progression of GBM.

Podocalyxin (PODX) is a highly glycosylated and sialylated transmembrane protein, and a CD34 ortholog normally expressed on hematopoietc stem cells, hemangioblasts, vascular endothelial cells, podocytes, and a subset of neural progenitors [4]. The clinical significance of PODX in cancer progression has been investigated in many cancer types. PODXL expression is correlated with tumor grade in uterine endometrioid adenocarcinoma [5]. Its overexpression is an independent indicator of poor outcome in breast and colorectal carcinoma [6], [7]. PODX also reportedly enhance in vitro invasion in breast cancer and prostate cancer cells [8]. A recent report has shown that PODX promotes astrocytoma cell invasion and survival against apoptotic stress [9], suggesting that PODX also contributes to GBM progression.

β-Catenin (β-cat), originally identified as an essential regulator for E-cadherin-mediated cell-cell interaction, is a key component of the Wnt signaling pathway [10]. In most cells, β-cat is predominantly located at the plasma membrane in a complex with cadherins and α-catenin, which is resistant to mild detergent such as Triton X-100 and Nonidet P-40. This is the insoluble pool of β-catenin. Under normal conditions, small amount of soluble β-cat is present in the cytoplasm free from cadherin [11]. Wnt signals are transduced via specific cell surface receptors to activate a series of biochemical reactions involving a large protein complex consisting of β-catenin and glycogen synthase kinase-3β (GSK-3β), resulting in stabilization of soluble β-cat and therefore an increase in the soluble pool of β-cat [12]. The soluble β-cat interacts with the T cell factor (Tcf) family transcription factors to activate a number of downstream target genes such as c-Myc and c-Jun, which play important roles in the progression of cancers [11], [13], [14]. Increased β-cat signaling has been linked to progression of a variety of cancers, including prostate cancer, hepatocarcinoma and renal cell carcinoma [14]–[16]. Recent studies have suggested that β-cat signaling is a key contributor to the proliferation and invasiveness of GBM cells [17], [18].

Apparently, both PODX and β-cat signaling play important roles in GBM progression. Our pilot study suggested that PODX could regulate β-cat signaling in GBM cells. In this study, we for the first time explored crosstalk between PODX and β-cat signaling in GBM cells, and assessed its impact on GBM cell invasion and proliferation.

## Materials and Methods

### Cells lines and reagents

LN-229 (CRL-2611) and U-118 MG (HTB-15) human GBM cell lines were purchased from the American Type Culture Collection (Manassas, VA, USA). Human full length PODX cDNA was subcloned into pcDNA 3.1 expression vector. Human PODX shRNA plasmid (RHS3979-98487921) was purchased from Open Biosystems (Huntsville, AL, USA). Human β-cat cDNA clone (SC107921) was purchased from from Origene (Beijing, China) and the β-cat cDNA sequence lacking those encoding 151 amino-terminal residues was subcloned into pcDNA 3.1 to generate a constitutively active (ΔN151) β-cat expression vector. Anti-PODX (3D3) (39-3800) antibody and Lipofectamine 2000 transfection reagent were purchased from Life Technologies (Carlsbad, CA, USA). Anti-β-cat (C-18) (sc-1496) (epitope matched to the carboxyl terminal of human β-cat), anti-matrix metalloproteinase 9 (MMP9) (M-17) (sc-6841) and anti-glyceraldehyde-3-phosphate dehydrogenase (GAPDH) (V-18) (sc-20357) antibodies were purchased from Santa Cruz Biotechnology (Santa Cruz, CA, USA). The anti-GSK-3β antibody was purchased from Cell Signaling Technology (Beverly, MA, USA). The anti-phospho-GSK-3β (serine 389) antibody was purchased from Millipore (Billerica, MA, USA). The SensoLyte 520 MMP-9 Assay Kit (#71155) was purchased from AnaSpec (Fremont, CA, USA). The Methylthiazoletetrazolium (MTT) Cell Proliferation Assay kit (ATCC #30-1010K) was purchased from American Type Culture Collection. The p38 mitogen-activated protein kinase (MAPK) Assay kit (#9820) was purchased from Cell Signaling Technology. Selective β-cat signaling inhibitor CCT031374 was purchased from Tocris Bioscience (Bristol, UK). Dual-luciferase reporter assay system was purchased from Promega (Madison, WI, USA). Puromycin, G418, and selective p38 mitogen-activated protein kinase (MAPK) inhibitor PD169316 were purchased from Sigma-Aldrich (St. Louis, MO, USA).

### Transfection and lentiviral transduction

The PODX and the constitutively active (ΔN151) β-cat expression vectors were respectively transfected into LN-229 and U-118 MG cells using Lipofectamine 2000 transfection reagent (Life Technologies) according to the manufacturer’s instructions. Pools of stable transfectants were generated via selection with G418 (800 µg/mL) by the manufacturer’s protocol. Lentiviral transduction was performed in LN-229 and U-118 MG cells. Lentiviral particles were packaged with vector psPAX2 and vector pMD2.G according to the manufacturer’s instructions (Open Biosystems). A control virus containing a scrambled shRNA sequence that would not lead to specific degradation of any cellular mRNA was used as a negative control for PODX-shRNA lentiviral particles. Pools of stable transductants were generated via selection with puromycin (5 µg/mL).

### Western blot analysis

In Western blot analyses, for whole cell lysates, cells were lysed in 250 µL of 2×SDS loading buffer (62.5 mM TrisHCl, pH 6.8, 2% SDS, 25% glycerol, 0.01% bromphenol blue, 5% 2-mercaptoethanol), and incubated at 95°C for 10 min. For soluble β-cat detection, cells were lysed in 0.1% Nonidet P-40 lysis buffer (0.1% Nonidet P-40, 10 mM HEPES, pH 7.5, 142.5 mM KCl, 5 mM MgCl_2_, and 1 mM EGTA). The lysates were centrifuged at 14,000×g for 10 min, and the supernatants were saved as soluble cell lysate [14]. Equal amount of proteins for each sample were separated by 8–15% SDS-polyacrylamide gel and blotted onto a polyvinylidene difluoride microporous membrane (Millipore). Membranes were incubated for 1 hour with a 1∶1000 dilution of primary antibody, and then washed and revealed using secondary antibodies with horseradish peroxidase conjugate (1∶5000, 1 hour). Peroxidase was revealed with a GE Healthcare ECL kit (Shanghai, China). Proteins were quantified before being loaded onto the gel.

### Real-time quantitative reverse transcription PCR

RNA was prepared from cells using TRIzol reagent (Life Technologies) followed by purification with TURBO DNA-free system (Ambion, Austin, TX, USA). The cDNAs were synthesized using SuperScript II reverse transcriptase (Life Technologies). Real-time quantitative PCR was performed on the LightCycler thermal cycler system (Roche Diagnostics, Indianapolis, IN, USA) using SYBR Green Mix (Life Technologies) as described by the manufacturer. The results were normalized against that of GAPDH in the same sample. The primers used are as follows: for β-cat (primers designed to measure both wild type β-cat and constitutively active ΔN151 β-cat mRNA levels), 5′- GATCATGCTAGCATGGCAATTCCTGAG-3′ (forward) and 5′- AAGATCGGTACCTCAGTTATCTACAGG-3′ (reverse); for c-Myc, 5′-GGACGACGAGACCTTCATCAA-3′ (forward) and 5′-CCAGCTTCTCTGAGACGAGCTT-3′ (reverse); for c-Jun, 5′-CAAAGTTTGGATTGCATCAAGTG-3′ (forward) and 5′-TAACATTATAAATGGTCACAGCACATG-3′ (reverse); for MMP9, 5′-GTTCCCGGAGTGAGTTGA-3′ (forward) and 5′-TTTACATGGCACTGCAAAGC-3′ (reverse); for GAPDH, 5′-GACTCATGACCACAGTCCATGC-3′ (forward) and 5′-AGAGGCAGGGATGATGTTCTG-3′ (reverse). Each experiment was repeated for three times in duplicates.

### Luciferase Assay

LN-229 and U-118 MG cells were transfected with TOPflash or FOPflash plasmids using Lipofectamine 2000 transfection reagent (Life Technologies). Plasmid PRL-CMV encoding *Renilla reniformis* luciferase (at one fifth molar ratio to test plasmids) was co-transfected in each transfection as an internal control for data normalization. The luciferase assays were performed 30 hours after transfection with a dual-luciferase reporter assay system (Promega) according to the manufacturer’s instructions. Each experiment was repeated for three times in duplicates.

### Cell invasion assay

Transwell cell-culture chambers with 8-µm pore size (BD Biosciences, Bedford, MA, USA) for 24-well plates were coated with 50 µL Matrigel (10 mg/mL; BD Biosciences) (diluted 1∶3). LN-229 and U-118 MG cells were seeded in the upper chamber at 3×10^4^ cells per well in RPMI 1640 serum-free medium. Complete medium (600 µL) with 10% fetal bovine serum was added to the lower chamber. After 30 hours of incubation, cells were fixed and stained with crystal violet. Invasion cells were counted in four random fields per chamber under microscope. Each experiment was repeated for three times in duplicates.

### MMP9 activity assay

MMP9 activity was measured with the SensoLyte 520 MMP-9 Assay Kit (AnaSpec) according to the manufacturer’s instructions [19], [20]. The supernatants were collected and then incubated with 4-aminophenylmercuric acetate (AMPA) and MMP9 substrate. The fluorescence intensity at Ex/Em Wave lengths of 490 nm/520 nm were used as a measure of MMP9 activity. Each experiment was repeated for three times in duplicates.

### MTT cell proliferation assay

In vitro cell proliferation was determined with a MTT Cell Proliferation Assay kit as described by the manufacturer (ATCC). Briefly, cells were cultured at 15×10^3^ cells per well in 96-well tissue culture plates and incubated at 37°C for 15 or 30 hours. At the end of the culture period, cells were washed with phosphate-buffered saline, the MTT reagents were added according to the manufacturer's recommendations, and the absorbance was measured at 570 nm using an ELISA plate reader. Each experiment was repeated for three times in triplicates.

### p38 MAPK activity assay

p38 MAPK activity was measured with the p38 MAPK Assay kit (Cell Signaling Technology) according to the manufacturer’s instructions [21]. Briefly, cells were directly lysed in the culture dishes. Cell lysates were sonicated and centrifuged at 15,000 rpm for 10 minutes at 4°C. The supernatant containing equivalent amounts of protein (200 µg) was incubated by gentle rocking with 20 µL of immobilized phospho-p38-MAPK monoclonal antibody for 16 hours at 4°C. The immunoprecipitates were washed twice with the lysing buffer and pelleted by centrifugation. The p38 MAPK assay was carried out using ATF2 fusion protein (2 µg) as a substrate in the presence of 200 µM ATP and 1×kinase buffer following the manufacturer’s recommendations. Samples were resolved on a 12% SDS-PAGE gel and visualized by autoradiography.

### Statistical analysis

Statistical analyses were performed with SPSS for Windows 10.0 (SPSS Inc., Chicago, IL, USA). All data values were expressed as means±SD. Comparisons of means among multiple groups were performed with one-way ANOVA followed by *post hoc* pairwise comparisons using Tukey’s tests. A two-tailed *p*<0.05 was considered statistically significant in this study.

## Results

### Effects of PODX on the protein levels of β-cat in GBM cells

**Figure 1:**
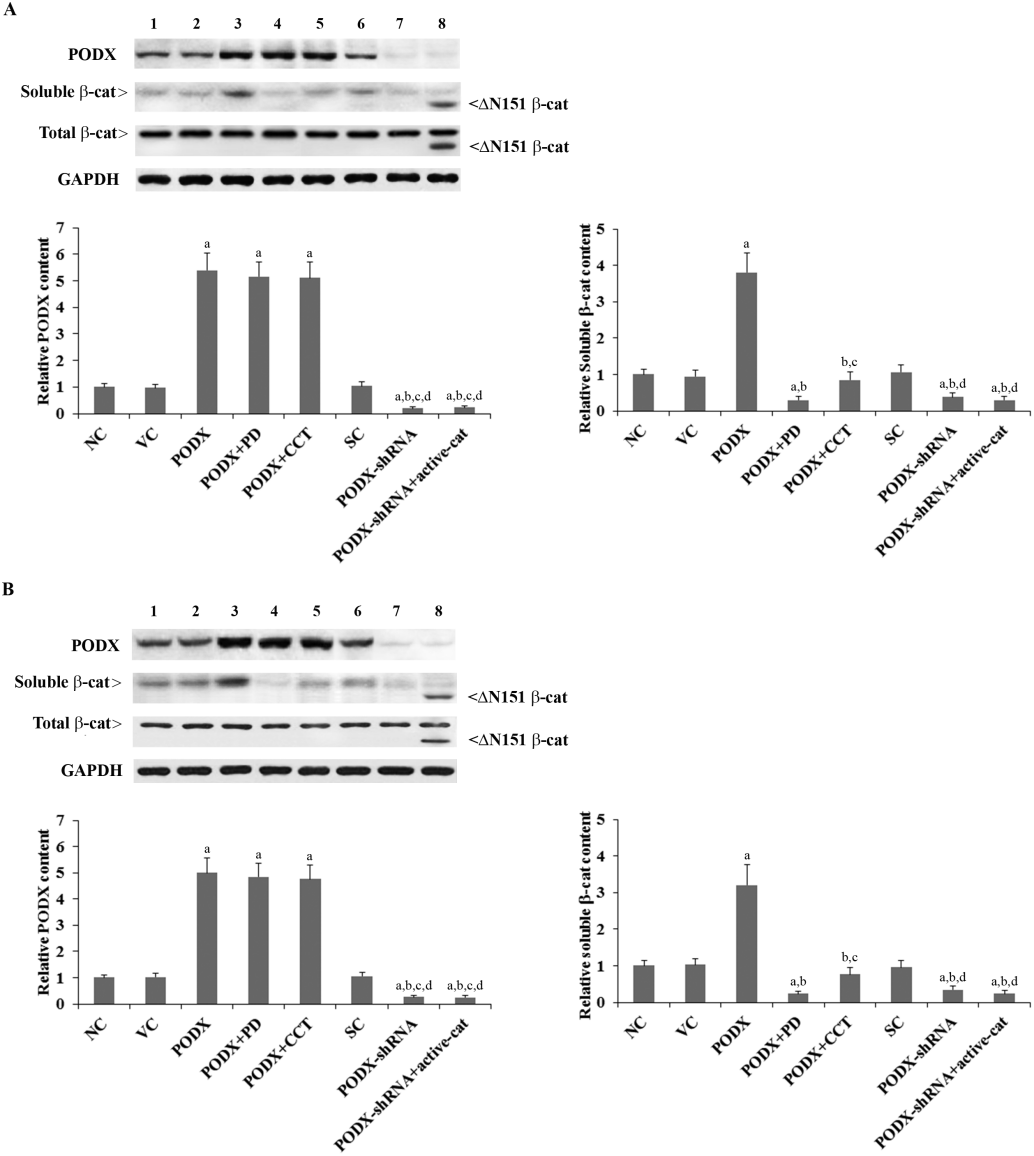
Protein levels of podocalyxin (PODX) and β-catenin (β-cat) in glioblastoma multiforme (GBM) cells with overexpression or knockdown of PODX. In (*A*) LN-229 and (*B*) U-118 MG human GBM cells, protein levels of PODX and soluble and total β-cat were determined with western blot analyses in normal control cells (NC, lane 1), cells stably transfected with the empty pcDNA3.1 vector (VC, lane 2), cells stably transfected with PODX (lane 3), cells stably transfected with PODX and treated with selective p38 mitogen-activated protein kinase (MAPK) inhibitor PD169316 (25 µM) for 30 hours (PODX+PD, lane 4), cells stably transfected with PODX and treated with selective β-cat signaling inhibitor CCT031374 (50 µM) for 30 hours (PODX+CCT, lane 5), cells stably transduced with scramble control shRNA (SC, lane 6), cells stably transduced with PODX-shRNA (lane 7), and cells stably transduced with PODX-shRNA and stably transfected with constitutively active (ΔN151) β-cat (PODX-shRNA+active-cat, lane 8). Glyceraldehyde-3-phosphate dehydrogenase (GAPDH) blotting was used as a loading control. The total β-cat protein level was not significantly altered by PODX in both LN-229 and U-118 MG cells. Density of the PODX and the soluble β-cat blots was normalized against that of the GAPDH blot to obtain a relative blot density, which was expressed as fold changes to that of NC (designated as 1) to represent the relative protein content. Three independent experiments were performed for each Western blot analysis. Data values were expressed as Mean+SD. ^a^ *p*<0.05 vs. controls (NC, VC and SC); ^b^ *p*<0.05 vs. PODX; ^c^ *p*<0.05 vs. PODX+PD; ^d^ *p*<0.05 vs. PODX+CCT.

We stably overexpressed PODX in LN-229 and U-118 MG human GBM cells by stable transfection, and on the other hand stably transduced the cells with lentiviral shRNA to knock down PODX. CCT031374, a selective β-cat signaling inhibitor that decreases the intracellular/soluble β-cat level [22], was employed to inhibit β-cat signaling in cells overexpressing PODX. A constitutively active β-cat mutant, which lacks 151 amino-terminal residues (ΔN151), was stably overexpressed in cells expressing PODX-shRNA. In addition, as our pilot study had suggested that PODX could regulate the soluble β-cat level in GBM cells by a p38 MAPK-dependent mechanism (data not shown), we included a selective p38 MAPK inhibitor PD169316 in all experiments in this study [23]. As shown in Fig. 1, compared with the controls, PODX was overexpressed 5.4 and 5 folds in LN-229 and U-118 MG cells, respectively; on the other hand, the endogenous PODX level was knocked down approximately 80% and 75% in LN-229 and U-118 MG cells, respectively. While the total β-cat protein level was not significantly altered by overexpression and knockdown of PODX, overexpression of PODX increased the soluble β-cat level by 3.8 folds in LN-229 cells and by 3.2 folds in U-118 MG cells, which was abolished by PD169316 and CCT031374. On the other hand, knockdown of PODX decreased the soluble β-cat level by over 60% in both cell lines. Fig. 1 also shows that the constitutively active (ΔN151) form of β-cat was overexpressed in the cells. It is apparent that CCT031374 and ΔN151 β-cat showed no significant effect on the protein level of PODX (Fig. 1), suggesting that inhibition or activation of β-cat signaling had no reciprocal effects on PODX expression.

### Effects of PODX on transcriptional activities of β-cat in GBM cells

**Figure 2:**
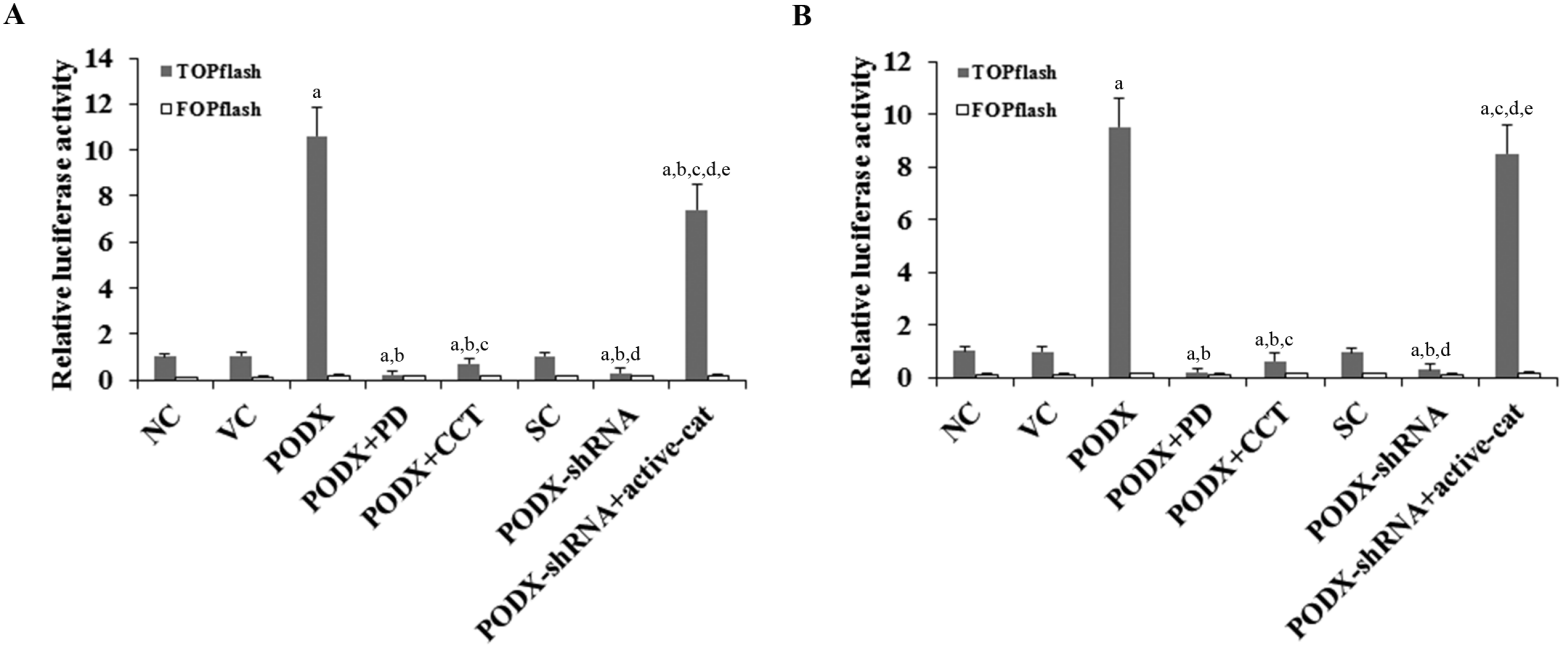
Effects of podocalyxin (PODX) on β-catenin (β-cat) signaling luciferase reporter activities in glioblastoma multiforme (GBM) cells. (*A*) LN-229 and (*B*) U-118 MG GBM cells were transfected with TOPflash, a synthetic β-catenin luciferase reporter, or FOPflash, a negative control reporter for TOPflash. Thirty hours later, the luciferase activity was determined in normal control cells (NC), cells stably transfected with the empty pcDNA3.1 vector (VC), cells stably transfected with PODX, cells stably transfected with PODX and treated with selective p38 mitogen-activated protein kinase (MAPK) inhibitor PD169316 (25 µM) for 30 hours (PODX+PD), cells stably transfected with PODX and treated with selective β-cat signaling inhibitor CCT031374 (50 µM) for 30 hours (PODX+CCT), cells stably transduced with scramble control shRNA (SC), cells stably transduced with PODX-shRNA, and cells stably transduced with PODX-shRNA and stably transfected with constitutively active (ΔN151) β-cat (PODX-shRNA+active-cat). The luciferase activity was expressed as fold changes to that of NC (designated as 1). ^a^ *p*<0.05 vs. controls (NC, VC and SC); ^b^ *p*<0.05 vs. PODX; ^c^ *p*<0.05 vs. PODX+PD; ^d^ *p*<0.05 vs. PODX+CCT; ^e^ *p*<0.05 vs. PODX-shRNA.

**Figure 3:**
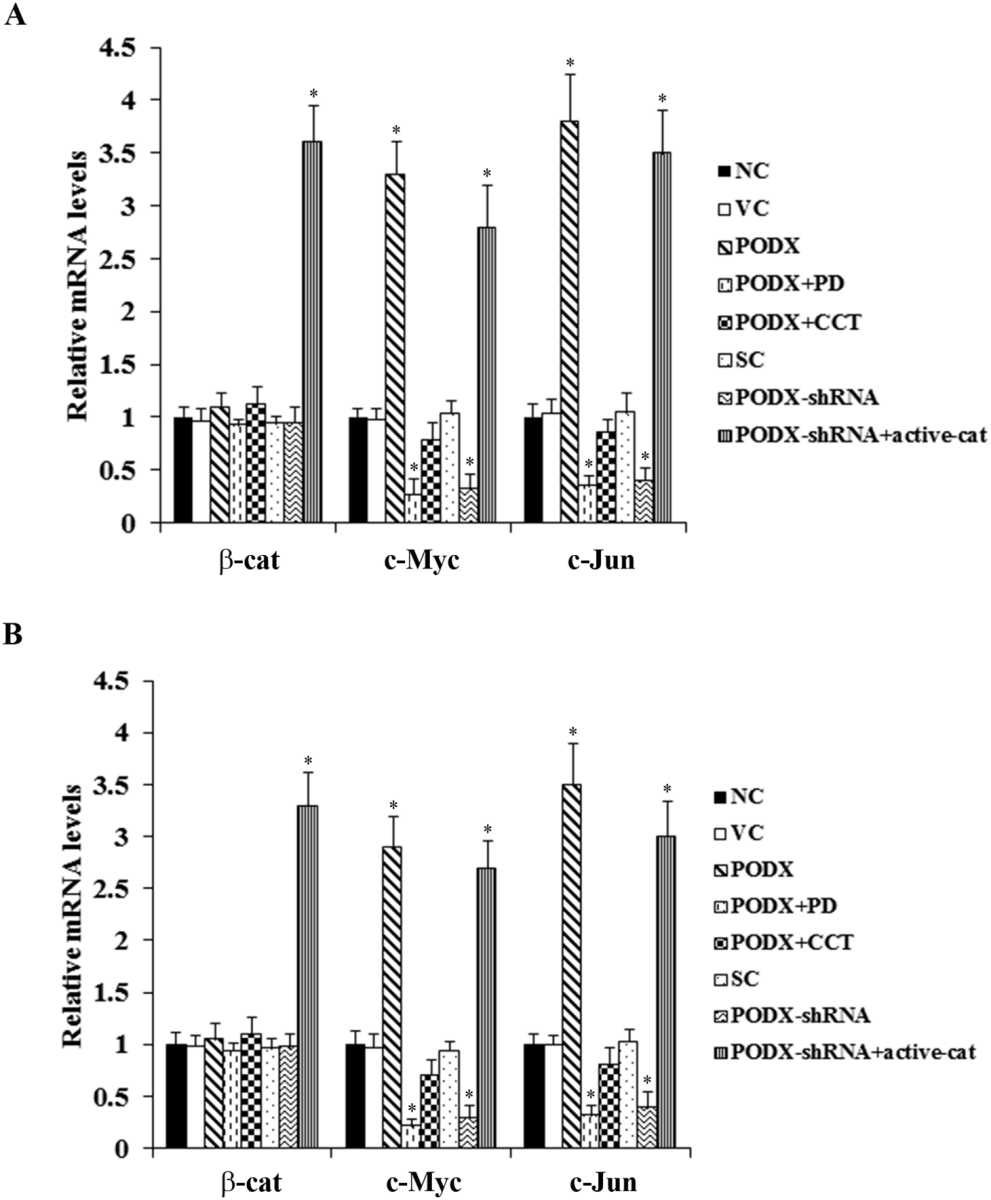
Effect of podocalyxin (PODX) on mRNA levels of β-catenin (β-cat), C-Myc and C-Jun in glioblastoma multiforme (GBM) cells. The mRNA levels of β-cat and β-cat signaling target genes C-Myc and C-Jun were determined in (*A*) LN-229 and (*B*) U-118 MG GBM cells. Real-time RT-PCR was performed with normal control cells (NC), cells stably transfected with the empty pcDNA3.1 vector (VC), cells stably transfected with PODX, cells stably transfected with PODX and treated with selective p38 mitogen-activated protein kinase (MAPK) inhibitor PD169316 (25 µM) for 30 hours (PODX+PD), cells stably transfected with PODX and treated with selective β-cat signaling inhibitor CCT031374 (50 µM) for 30 hours (PODX+CCT), cells stably transduced with scramble control shRNA (SC), cells stably transduced with PODX-shRNA, and cells stably transduced with PODX-shRNA and stably transfected with constitutively active (ΔN151) β-cat (PODX-shRNA+active-cat). The mRNA level was expressed as fold changes to that of NC (designated as 1). *p<0.05 vs. controls (NC, VC and SC).

As the above results suggested that PODX could significantly elevate the soluble β-cat level in GBM cells, we next examined whether PODX would activate β-cat signaling in GBM cells. As shown in Fig. 2, transcriptional activities of β-cat in LN-229 and U-118 MG cells were measured with TOPflash, a synthetic β-cat/Tcf-dependent luciferase reporter [14]. Compared with controls, overexpression of PODX increased the luciferase activity of TOPflash by 10.6 folds in LN-229 cells and by 9.5 folds in U-118 MG cells, which was abolished by PD169316 and CCT031374. On the other hand, knockdown of PODX decreased the luciferase activity of TOPflash by approximately 70% in both cell lines, which was completely reversed by overexpression of ΔN151 β-cat. Little change was observed with FOPflash, a negative control reporter with mutated Tcf binding elements (Fig. 2) [14]. As shown in Fig. 3, real-time RT-PCR showed that overexpression or knockdown of PODX did not significantly affect β-cat mRNA levels in LN-229 and U-118 MG cells. However, the mRNA levels of β-cat signaling target genes (c-Myc and c-Jun) were increased over 3.3 folds by overexpression of PODX, which was abolished by PD169316 and CCT031374. Knockdown of PODX decreased the mRNA levels of c-Myc and c-Jun by over 60% in both cell lines, which was completely reversed by overexpression of ΔN151 β-cat (Fig. 3). Taken together, the results indicate that PODX can activate β-cat signaling in GBM cells by post-transcriptionally elevating the soluble β-cat level through a p38 MAPK-dependent mechanism. In addition, real-time RT-PCR with primers specifically designed to measure both wild type β-cat and ΔN151 β-cat mRNA levels showed that stable transfection of ΔN151 β-cat markedly increased the detected β-cat mRNA level, which was in agreement with the strong ΔN151 β-cat protein expression in Fig. 1.

### Effects of PODX/β-cat signaling on cell invasion and MMP9 expression/activity in GBM cells

**Figure 4:**
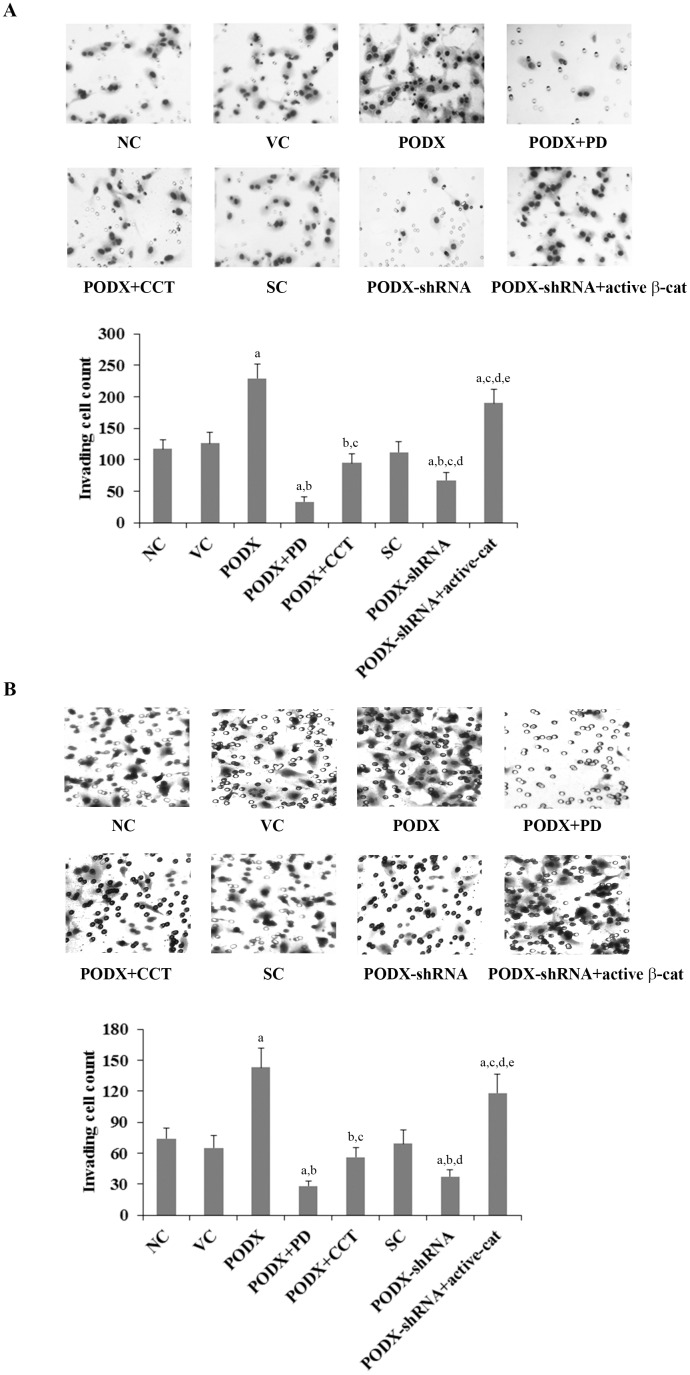
Effects of podocalyxin (PODX)/β-catenin (β-cat) signaling on glioblastoma multiforme (GBM) cell invasion. In vitro cell invasion assays were performed in (*A*) LN-229 and (*B*) U-118 MG GBM cells. Invading cells were fixed and counted in normal control cells (NC), cells stably transfected with the empty pcDNA3.1 vector (VC), cells stably transfected with PODX, cells stably transfected with PODX and treated with selective p38 mitogen-activated protein kinase (MAPK) inhibitor PD169316 (25 µM) for 30 hours (PODX+PD), cells stably transfected with PODX and treated with selective β-cat signaling inhibitor CCT031374 (50 µM) for 30 hours (PODX+CCT), cells stably transduced with scramble control shRNA (SC), cells stably transduced with PODX-shRNA, and cells stably transduced with PODX-shRNA and stably transfected with constitutively active (ΔN151) β-cat (PODX-shRNA+active-cat). Representative cell invasion images are shown. ^a^ *p*<0.05 vs. controls (NC, VC and SC); ^b^ *p*<0.05 vs. PODX; ^c^ *p*<0.05 vs. PODX+PD; ^d^ *p*<0.05 vs. PODX+CCT; ^e^ *p*<0.05 vs. PODX-shRNA.

To examine effects of the PODX/β-cat signaling axis on GBM cell invasion, we performed in vitro cell invasion assays. Compared with the controls, overexpression of PODX increased cell invasion by approximately 2 folds in LN-229 and U-118 MG cells, which was abolished by PD169316 and CCT031374 (Fig. 4). On the other hand, knockdown of PODX decreased cell invasion by approximately 50% in both cell lines, which was completely reversed by overexpression of ΔN151 β-cat (Fig. 4).

**Figure 5:**
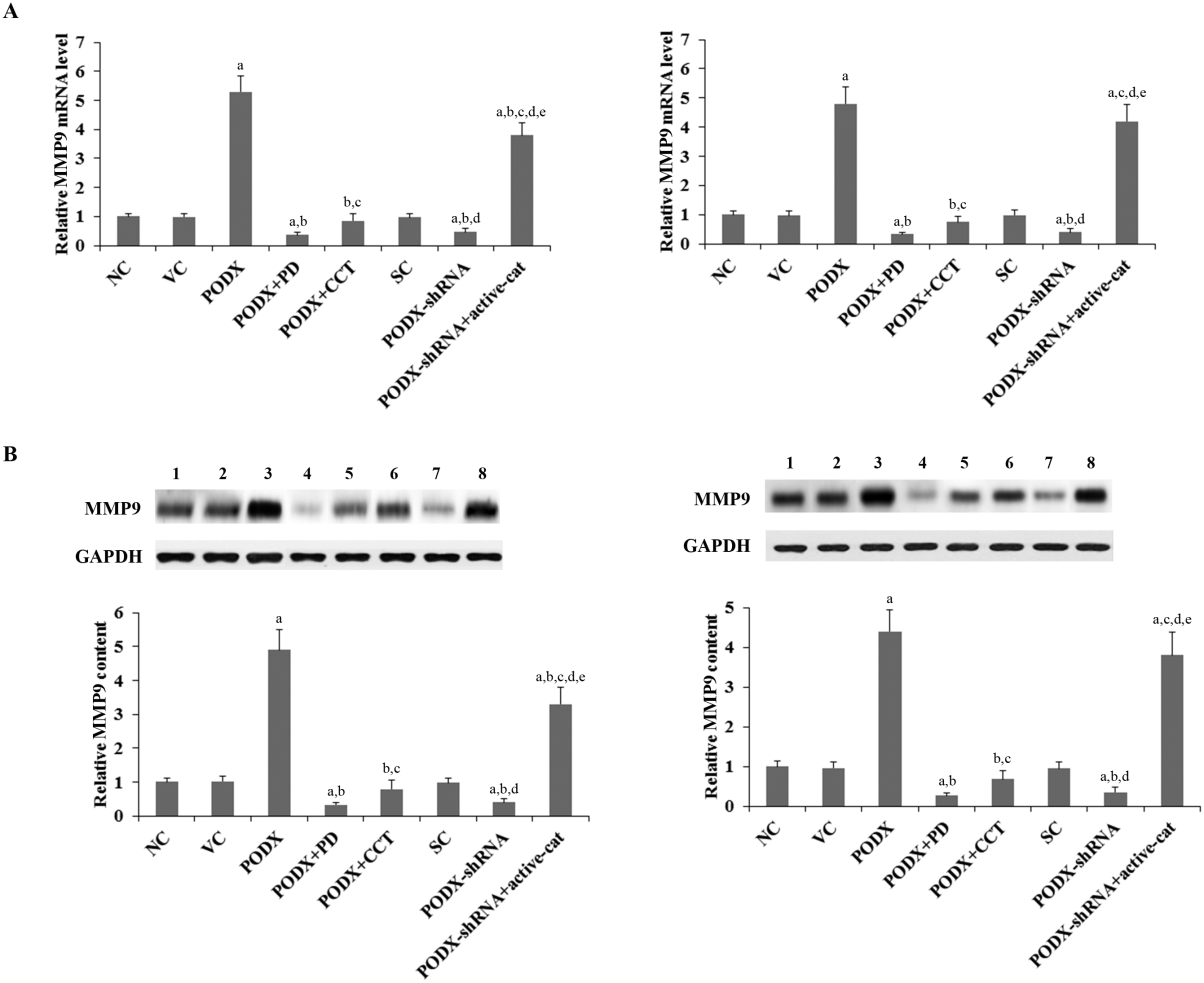
Effects of podocalyxin (PODX)/β-catenin (β-cat) signaling on expression of matrix metalloproteinase 9 (MMP9) in glioblastoma multiforme (GBM) cells. The (*A*) mRNA and the (*B*) protein levels of MMP9 in LN-229 (*left panel*) and U-118 MG (*right panel*) GBM cells were respectively determined by real-time RT-PCR and Western blot analyses in normal control cells (NC, lane 1), cells stably transfected with the empty pcDNA3.1 vector (VC, lane 2), cells stably transfected with PODX (lane 3), cells stably transfected with PODX and treated with selective p38 mitogen-activated protein kinase (MAPK) inhibitor PD169316 (25 µM) for 30 hours (PODX+PD, lane 4), cells stably transfected with PODX and treated with selective β-cat signaling inhibitor CCT031374 (50 µM) for 30 hours (PODX+CCT, lane 5), cells stably transduced with scramble control shRNA (SC, lane 6), cells stably transduced with PODX-shRNA (lane 7), and cells stably transduced with PODX-shRNA and stably transfected with constitutively active (ΔN151) β-cat (PODX-shRNA+active-cat, lane 8). The mRNA and the protein levels of MMP9 were expressed as fold changes to those of NC (designated as 1), respectively. Three independent experiments were performed for each Western blot analysis. Data values were expressed as Mean+SD. ^a^ *p*<0.05 vs. controls (NC, VC and SC); ^b^ *p*<0.05 vs. PODX; ^c^ *p*<0.05 vs. PODX+PD; ^d^ *p*<0.05 vs. PODX+CCT; ^e^ *p*<0.05 vs. PODX-shRNA.

**Figure 6:**
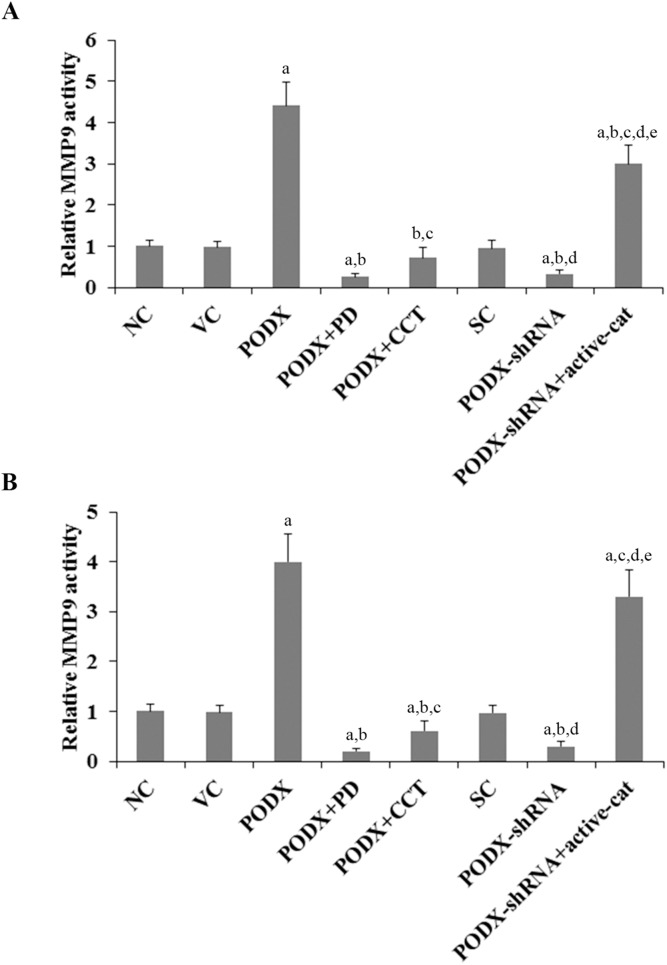
Effects of podocalyxin (PODX)/β-catenin (β-cat) signaling on matrix metalloproteinase 9 (MMP9) activities in glioblastoma multiforme (GBM) cells. In (*A*) LN-229 and (*B*) U-118 MG GBM cells, MMP9 activities were determined with a SensoLyte 520 MMP9 Assay Kit (AnaSpec) in normal control cells (NC), cells stably transfected with the empty pcDNA3.1 vector (VC), cells stably transfected with PODX, cells stably transfected with PODX and treated with selective p38 mitogen-activated protein kinase (MAPK) inhibitor PD169316 (25 µM) for 30 hours (PODX+PD), cells stably transfected with PODX and treated with selective β-cat signaling inhibitor CCT031374 (50 µM) for 30 hours (PODX+CCT), cells stably transduced with scramble control shRNA (SC), cells stably transduced with PODX-shRNA, and cells stably transduced with PODX-shRNA and stably transfected with constitutively active (ΔN151) β-cat (PODX-shRNA+active-cat). The MMP9 activity was shown as fold changes to that of NC (designated as 1). ^a^ *p*<0.05 vs. controls (NC, VC and SC); ^b^ *p*<0.05 vs. PODX; ^c^ *p*<0.05 vs. PODX+PD; ^d^ *p*<0.05 vs. PODX+CCT; ^e^ *p*<0.05 vs. PODX-shRNA.

MMPs play a critical role in cancer cell invasion [24]. Among different MMPs tested, we found that the MMP9 expression was significantly altered by PODX/β-cat signaling in GBM cells. As shown in Fig. 5, compared with the controls, overexpression of PODX increased MMP9 expression at both the mRNA and the protein levels by over 4 folds in LN-229 and U-118 MG cells, which was abolished by PD169316 and CCT031374. Knockdown of PODX decreased MMP9 expression by approximately 60% in both cell lines, which was completely reversed by overexpression of ΔN151 β-cat (Fig. 5). Similar data trend was observed with the MMP9 activity (Fig. 6).

### Effects of PODX/β-cat signaling on GBM cell proliferation

**Figure 7:**
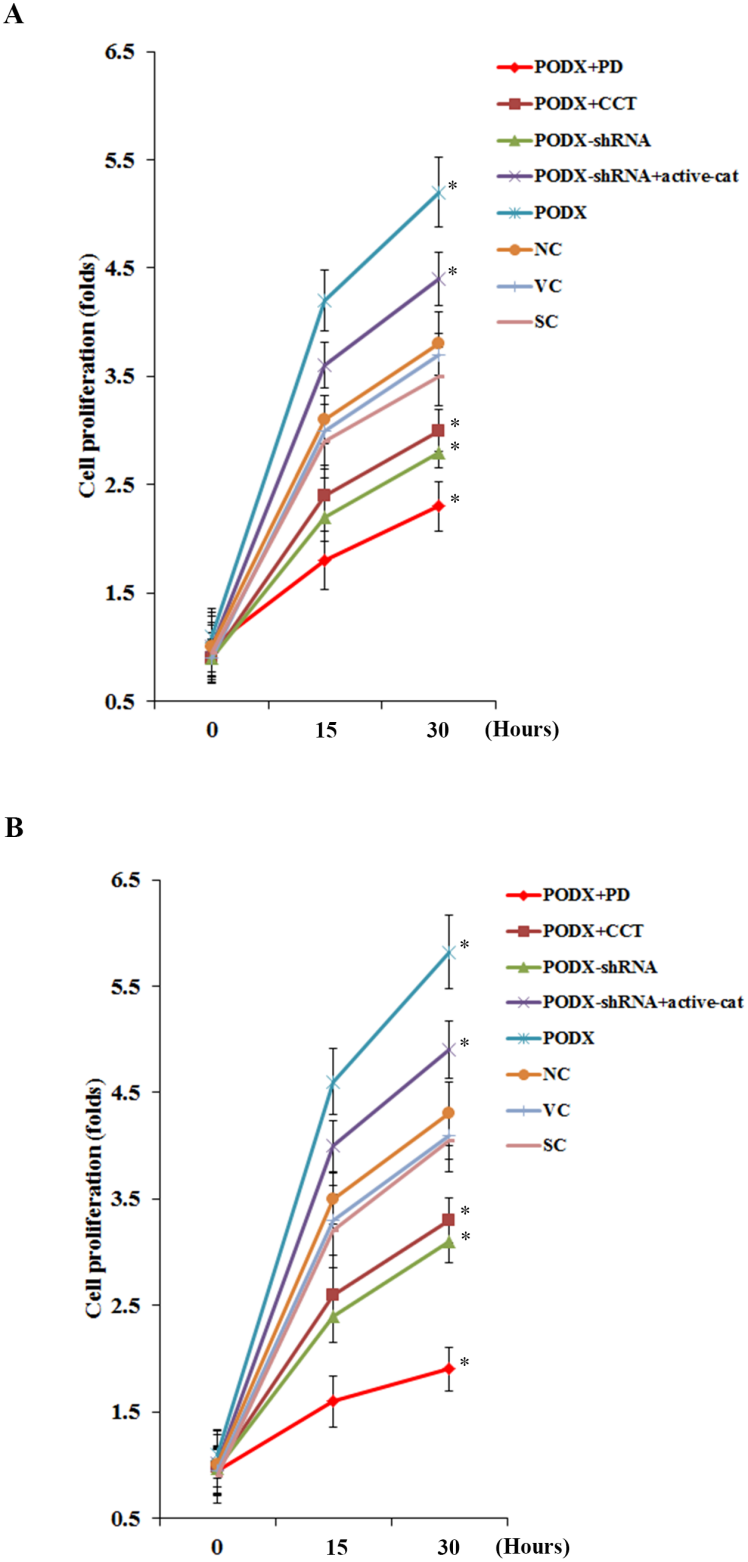
Effects of podocalyxin (PODX)/β-catenin (β-cat) signaling on glioblastoma multiforme (GBM) cell proliferation. In (*A*) LN-229 and (*B*) U-118 MG GBM cells, methlythiazoletetrazolium (MTT) cell proliferation assays were performed for 15 or 30 hours in normal control cells (NC), cells stably transfected with the empty pcDNA3.1 vector (VC), cells stably transfected with PODX, cells stably transfected with PODX and treated with selective p38 mitogen-activated protein kinase (MAPK) inhibitor PD169316 (25 µM) for 30 hours (PODX+PD), cells stably transfected with PODX and treated with selective β-cat signaling inhibitor CCT031374 (50 µM) for 15 or 30 hours (PODX+CCT), cells stably transduced with scramble control shRNA (SC), cells stably transduced with PODX-shRNA, and cells stably transduced with PODX-shRNA and stably transfected with constitutively active (ΔN151) β-cat (PODX-shRNA+active-cat). Cell proliferation at 15 and 30 hours was expressed as fold changes to that of NC (designated as 1). *p<0.05 vs. controls (NC, VC and SC) at 30 hours.

To examine effects of the PODX/β-cat signaling axis on GBM cell proliferation, we performed MTT cell invasion assays. Compared with the controls, overexpression of PODX significantly increased cell proliferation in LN-229 and U-118 MG cells after 30 hours of culture, which was abolished by PD169316 and CCT031374 (Fig. 7). On the other hand, knockdown of PODX significantly inhibited cell proliferation in both cell lines, which was completely reversed by overexpression of ΔN151 β-cat (Fig. 7).

### Effects of PODX on p38 MAPK activities in GBM cells

**Figure 8:**
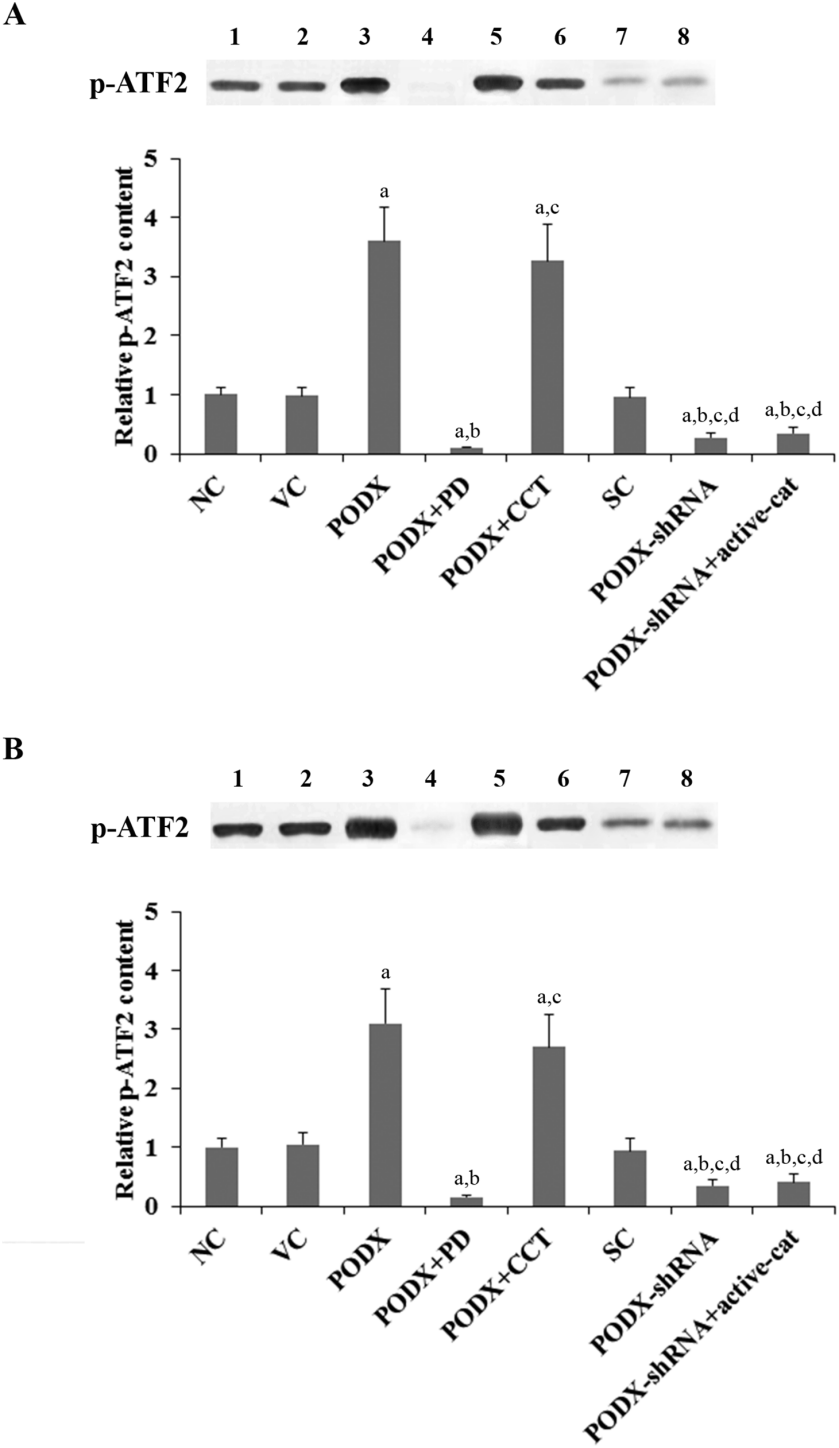
Effects of podocalyxin (PODX)/β-catenin (β-cat) signaling on p38 mitogen-activated protein kinase (MAPK) activities in glioblastoma multiforme (GBM) cells. In (*A*) LN-229 and (*B*) U-118 MG GBM cells, the p38 MAPK activity was determined with a p38 MAPK Assay kit (Cell Signaling Technology) by measuring phosphorylation of ATF2, a substrate of activated p38 MAPK. The levels of phosphorylated ATF2 (p-ATF2) were determined with western blot in normal control cells (NC, lane 1), cells stably transfected with the empty pcDNA3.1 vector (VC, lane 2), cells stably transfected with PODX (lane 3), cells stably transfected with PODX and treated with selective p38 mitogen-activated protein kinase (MAPK) inhibitor PD169316 (25 µM) for 30 hours (PODX+PD, lane 4), cells stably transfected with PODX and treated with selective β-cat signaling inhibitor CCT031374 (50 µM) for 30 hours (PODX+CCT, lane 5), cells stably transduced with scramble control shRNA (SC, lane 6), cells stably transduced with PODX-shRNA (lane 7), and cells stably transduced with PODX-shRNA and stably transfected with constitutively active (ΔN151) β-cat (PODX-shRNA+active-cat, lane 8). The p-ATF2 content/p38 MAPK activity was shown as fold changes to that of NC (designated as 1). Each experiment was repeated for three times in duplicates. Data values were expressed as Mean+SD. ^a^ *p*<0.05 vs. controls (NC, VC and SC); ^b^ *p*<0.05 vs. PODX; ^c^ *p*<0.05 vs. PODX+PD; ^d^ *p*<0.05 vs. PODX+CCT.

The above results suggested that PODX could stimulate GBM cell invasion and proliferation by promoting β-cat signaling though a p38-dependent mechanism. Therefore, we next examined effects of the PODX/β-cat signaling axis on p38 MAPK activity, which was measured by phosphorylation of ATF2, a substrate of activated p38 MAPK [21]. As evidenced by increased expression of phosphorylated ATF2, overexpression of PODX respectively induced p38 MAPK activity by 3.6 and 3.1 folds in LN-229 and U-118 MG cells, which was abolished by PD169316, but not CCT031374 (Fig. 8). On the other hand, knockdown of PODX decreased p38 MAPK activity by approximately 70% in both cell lines, which was not significantly affected by overexpression of ΔN151 β-cat (Fig. 8). The results indicate that PODX and β-cat signaling are respectively upstream and downstream of p38 MAPK in the PODX/β-cat signaling axis.

### Effects of PODX on inactivating phosphorylation of GSK-3β in GBM cells

**Figure 9:**
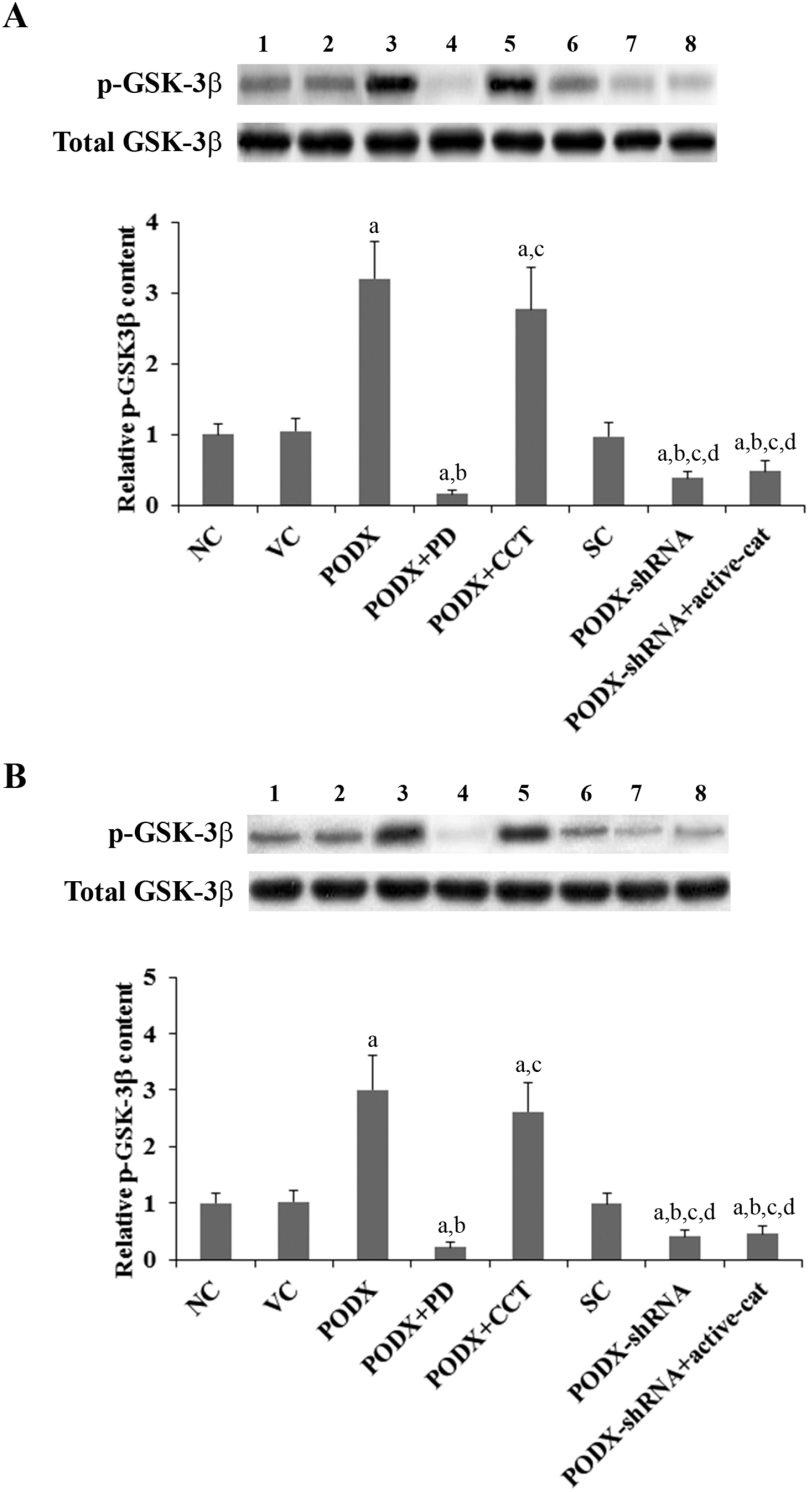
Effects of podocalyxin (PODX)/β-catenin (β-cat) signaling on phosphorylated glycogen synthase kinase-3β (p-GSK-3β) levels in glioblastoma multiforme (GBM) cells. In (*A*) LN-229 and (*B*) U-118 MG GBM cells, the levels of p-GSK-3β at serine 389 and total GSK-3β were determined with Western blot analyses in normal control cells (NC, lane 1), cells stably transfected with the empty pcDNA3.1 vector (VC, lane 2), cells stably transfected with PODX (lane 3), cells stably transfected with PODX and treated with selective p38 mitogen-activated protein kinase (MAPK) inhibitor PD169316 (25 µM) for 30 hours (PODX+PD, lane 4), cells stably transfected with PODX and treated with selective β-cat signaling inhibitor CCT031374 (50 µM) for 30 hours (PODX+CCT, lane 5), cells stably transduced with scramble control shRNA (SC, lane 6), cells stably transduced with PODX-shRNA (lane 7), and cells stably transduced with PODX-shRNA and stably transfected with constitutively active (ΔN151) β-cat (PODX-shRNA+active-cat, lane 8). The total GSK-3β protein level was not significantly altered by PODX in both LN-229 and U-118 MG cells. Density of the p-GSK-3β (serine 389) blot was normalized against that of the total GSK-3β blot to obtain a relative blot density, which was expressed as fold changes to that of NC (designated as 1). Three independent experiments were performed for each Western blot analysis. Data values were expressed as Mean+SD. ^a^ *p*<0.05 vs. controls (NC, VC and SC); ^b^ *p*<0.05 vs. PODX; ^c^ *p*<0.05 vs. PODX+PD; ^d^ *p*<0.05 vs. PODX+CCT.

GSK-3β reportedly is a downstream target of the p38 MAPK pathway [25], [26]. Phosphorylation of GSK-3β at serine 389 by p38 enhances inactivation of GSK-3β and results in subsequent stabilization and accumulation of soluble β-cat [11], [27]. Since our results had indicated that PODX could elevate the soluble β-cat level/β-cat signaling through p38 MAPK in GBM cells, we next examined effects of PODX on the level of inactivating phosphorylation of GSK-3β at serine 189 in GBM cells. As shown in Fig. 9, while the total GSK-3β was not significantly changed, overexpression of PODX respectively increased the level of phosphorylated GSK-3β (serine 389) by 3.2 and 3.0 folds in LN-229 and U-118 MG cells, which was abolished by PD169316, but not CCT031374. On the other hand, knockdown of PODX decreased phosphorylation of GSK-3β at serine 389 by approximately 60% in both cell lines, which was not significantly affected by overexpression of ΔN151 β-cat (Fig. 9). Changes in the level of inactivating phosphorylation of GSK-3β at serine 189 (Fig. 9) showed similar data trend to that of the p38 MAPK activity (Fig. 8) and in agreement with changes in the soluble β-cat level (Fig. 1). Taken together, the results indicate that PODX elevate the soluble β-cat level/β-cat signaling in GBM cells via the p38 MAPK/GSK-3β pathway.

## Discussion

Both PODX and β-cat has been found important for malignant progression in a variety of cancers [5]–[9], [14]–[16]. Using GBM cell models, we in the present study provide the first evidence supporting a crosstalk between PODX and β-cat signaling in cancer cells. We used two GBM cell lines as cell models in this study: (1) LN229, established from glioblastoma in the brain cortex of a 60-year-old female, showing epithelial morphology; (2) U-118 MG established from glioblastoma of a 50-year-old male, showing mixed morphology. Similar findings in the two cell models with relatively big background differences demonstrate a generalizable role of a PODX/β-cat signaling axis in GBM.

PODX is thought to regulate cell morphology and adhesion through its connections to intracellular proteins and to extracellular ligands [28]. β-cat is both a structural component of cell-cell contact sites and a signaling protein that activates the Wnt survival pathway [17], [18]. In the absence of Wnt ligands, cytoplasmic/soluble β-cat is constantly degraded by the axin complex. Wnt signals are transduced via specific cell surface receptors to deactivate the destruction complex, resulting in accumulation of cytoplasmic/soluble β-cat and transcriptional activation of β-cat/Tcf-regulated genes [17]. In this study, overexpression and knockdown of PODX in GBM cells respectively increased and decreased the soluble β-cat level without significantly altering the expression of β-cat at the mRNA and the total protein levels, suggesting PODX elevates the soluble β-cat level/β-cat signaling in GBM cells by a post-translational modification mechanism. In addition, as a selective p38 MAPK inhibitor readily abolished the PODX-elevated soluble β-cat level without significantly altering the expression of PODX and β-cat, it indicates that PODX elevates the soluble β-cat level/β-cat signaling in a post-translational p38 MAPK-dependent manner in GBM cells. The underlying mechanisms will be uncovered in our future studies.

PODX reportedly enhances invasion in many cancers, including GBM [5]–[9]. Although there is evidence that PODX participate in epithelial-mesenchymal transition and interacts with different mediators of metastasis, the role of PODX is not yet fully understood [28]. Abnormal activation of β-cat signaling plays a pivotal role in the progression of a variety of cancers [17]. Recent studies have suggested that β-cat signaling is a key contributor to the proliferation and invasiveness of GBM cells. In our study, overexpression of PODX significantly increased GBM cell invasion, which was abolished by a selective β-cat signaling inhibitor that decreases the soluble β-cat level [22], suggesting that β-cat signaling is major downstream mediator of the promoting effect of PODX on GBM cell invasion. This was corroborated by the finding that knockdown of PODX significantly inhibited GBM dell invasion, which was completely reversed by overexpression of a constitutively active β-cat mutant. GSK-3β activity is a determinant of β-cat stabilization and accumulation of soluble β-cat (Sharma et al., 2002), and p38 MAPK has been shown to inhibit GSK-3β activity via phosphorylating serine 389 [25]. In line with the findings, our study showed that PODX increased the level of phosphorylated GSK-3β at serine 389 as well as the soluble β-cat level via p38 MAPK in GBM cells.

Among different MMPs tested, we found that the MMP9 expression/activity was significantly altered by PODX/β-cat signaling, which is in agreement with a previous report that PODX induces MMP9 expression in GBM cells [9]. Our study has further revealed that β-cat signaling is a major mediator of PODX-induced MMP9 expression in GBM cells, for a selective β-cat signaling inhibitor abolished the promoting effect of PODX on MMP9 expression. This was confirmed by the finding that direct activation of β-cat signaling by overexpressing a constitutively active β-cat mutant completely reversed the inhibitory effects of PODX-knockdown on MMP9 expression. The inducing effect of PODX/β-cat signaling on MMP9 expression/activity may at least partially explain for its promoting effect on GBM cell invasion. In addition, our results show that PODX/β-cat signaling activates MMP9 expression at the mRNA transcription level. Future studies are needed to examine whether this is a direct effect of β-cat/Tcf transcriptional activities, or further downstream of activated target genes of β-cat/Tcf signaling.

Activation of β-cat signaling promote cell proliferation [17]. Indeed, our study has shown that PODX promotes GBM cell proliferation by elevating the soluble β-cat level/β-cat signaling. As the PODX/β-cat signaling axis enhances both invasion and proliferation of GBM cells, it may play an important role in GBM progression. In addition, since both PODX and β-cat are essential drivers of malignant progression in many cancers [5]–[9], [14]–[16], the PODX/β-cat signaling axis may also play important roles in the progression of other cancers besides GBM, which needs to be verified in future studies.

Like several other solid tumors, GBM are considered to be driven by a small sub-population of cells known as glioma stem cells [29]. β-cat signaling reportedly is essential for cancer stem cell maintenance [30]. PODX is also a well-known stem cell marker, and is closely related to stem cell marker CD34 and to endoglycan [25]. Thus, based on our findings, it will also be interesting to examine in future studies the role of the PODX/β-cat signaling axis in glioma stem cells.

In conclusion, our study indicates that PODX promotes GBM cell invasion and proliferation by elevating the soluble β-cat level/β-cat signaling through the p38 MAPK/GSK-3β pathway. Uncovering the PODX/β-cat signaling axis adds new insights not only into the biological functions of PODX and β-cat, but also into the molecular mechanisms underlying GBM progression.
