# Correction: Podocalyxin Promotes Glioblastoma Multiforme Cell Invasion and Proliferation via β-Catenin Signaling

**DOI:** 10.1371/journal.pone.0119049

**Published:** 2015-03-18

**Authors:** The PLOS ONE Staff

Two authors are missing from the author byline.

Dr. Liang Yang should be included in the author byline. Dr. Yang should be listed as the second author, and affiliated with:

2 Department of Neurosurgery, Third Xiangya Hospital, Central South University, Changsha, Hunan, China.

The contributions of this author are as follows: Conceived and designed the experiments, performed the experiments, analyzed the data.

Dr. Bo Liu should be included in the author byline. Dr. Liu should be listed as the third author, and affiliated with:

1 Department of Neurosurgery, Second Xiangya Hospital, Central South University, Changsha, Hunan, China

The contributions of this author are as follows: Performed the experiments, analyzed the data.

The correct citation is: Liu Y, Yang L, Liu B, Jiang Y-G (2014) Podocalyxin Promotes Glioblastoma Multiforme Cell Invasion and Proliferation via β-Catenin Signaling. PLoS ONE 9(10): e111343. doi:10.1371/journal.pone.0111343

## References

[1] Liu Y, Jiang Y-G (2014) Podocalyxin Promotes Glioblastoma Multiforme Cell Invasion and Proliferation via β-Catenin Signaling. PLoS ONE 9(10): e111343 doi:10.1371/journal.pone.0111343 25349993 10.1371/journal.pone.0111343 PMC4211695

